# Detection, Identification and Diagnostic Characterization of the Staphylococcal Small Colony-Variant (SCV) Phenotype

**DOI:** 10.3390/antibiotics12091446

**Published:** 2023-09-14

**Authors:** Karsten Becker

**Affiliations:** Friedrich Loeffler-Institute of Medical Microbiology, University Medicine Greifswald, Ferdinand-Sauerbruch-Straße 1, 17489 Greifswald, Germany; karsten.becker@med.uni-greifswald.de; Tel.: +49-3834-86-5560; Fax: +49-3834-86-5561

**Keywords:** small-colony-variant, *Staphylococcus aureus*, coagulase-negative staphylococci, diagnostics, cultivation, identification, auxotrophy, chronic infection, relapse, intracellular

## Abstract

While modern molecular methods have decisively accelerated and improved microbiological diagnostics, phenotypic variants still pose a challenge for their detection, identification and characterization. This particularly applies if they are unstable and hard to detect, which is the case for the small-colony-variant (SCV) phenotype formed by staphylococci. On solid agar media, staphylococcal SCVs are characterized by tiny colonies with deviant colony morphology. Their reduced growth rate and fundamental metabolic changes are the result of their adaptation to an intracellular lifestyle, regularly leading to specific auxotrophies, such as for menadione, hemin or thymidine. These alterations make SCVs difficult to recognize and render physiological, biochemical and other growth-based methods such as antimicrobial susceptibility testing unreliable or unusable. Therefore, diagnostic procedures require prolonged incubation times and, if possible, confirmation by molecular methods. A special approach is needed for auxotrophy testing. However, standardized protocols for SCV diagnostics are missing. If available, SCVs and their putative parental isolates should be genotyped to determine clonality. Since their detection has significant implications for the treatment of the infection, which is usually chronic and relapsing, SCV findings should be specifically reported, commented on, and managed in close collaboration with the microbiological laboratory and the involved clinicians.

## 1. Introduction

Staphylococci and other microorganisms are capable of forming a distinct phenotype referred to as the small-colony-variant (SCV) phenotype [1,2]. Most of the case reports and clinical studies on SCVs, as well as in vitro investigations, refer to *Staphylococcus aureus*; however, SCVs belonging to other staphylococcal species have also been recovered from clinical specimens including *S. argenteus* [3], *S. capitis* [4], *S. epidermidis* [4,5,6], *S. lugdunensis* [7,8], *S. pseudintermedius* [9] and *S. warneri* [6].

As the designation “small colony” or historical terms as “dwarf” or “gonidial” suggest [10,11,12], the colonies of SCVs on solid agar media are very small compared to the normal (wild) type even after prolonged incubation. The delayed growth of the SCVs is based on changes in the microorganism’s metabolism during its adaptation from normal extracellular occurrence to an intracellular lifestyle, which appears to be a dynamic integral part of the infection process, to hide in host cells [13,14]. Survival within nonprofessional phagocytes, such as epithelial and endothelial cells, fibroblasts, keratinocytes and osteoblasts, is a key characteristic of staphylococcal SCVs [1].

While the diagnostically relevant phenotypic features of SCVs appear to be quite uniform, the underlying mechanisms leading to the expression of the phenotype are very diverse and include alterations and dynamic regulation processes, e.g., in the electron transport chain or thymidine and fatty acid metabolism [15,16,17,18]. The resulting metabolic changes may affect other phenotypic traits that are classically used for identification, such as biochemical, physiological and antigenic features [1,19]. In addition, it impedes the antimicrobial susceptibility testing (AST) of SCVs [19].

**Figure 1:**
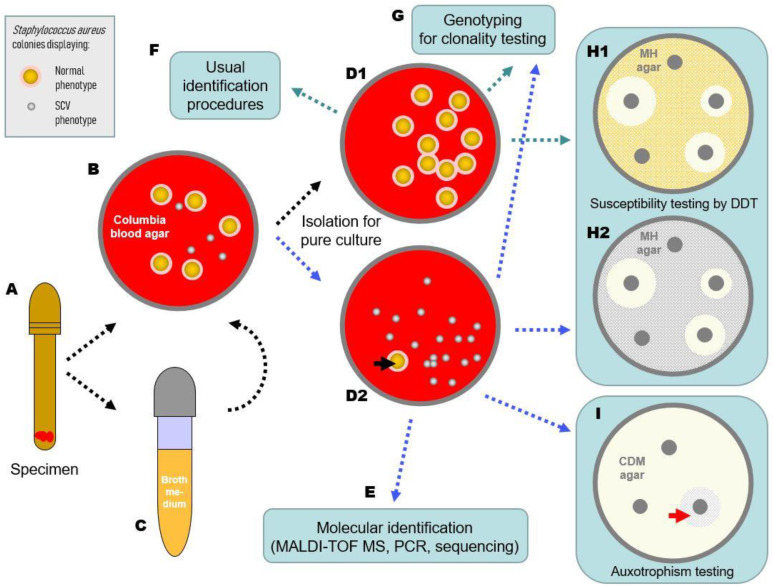
Flow scheme (dotted arrows) of the diagnostics of staphylococcal SCVs (exemplary for *S. aureus*). Subsequent to sample (e.g., tissue) collection (**A**) and inoculation on solid (**B**) and broth (**C**) media (to be striked out on agar plates after 48–72 h of incubation), agar plates should be inspected for the presence of SCV colonial morphotypes. In the case of mixed cultures, colonies displaying the normal phenotype (NP with yellowish pigmented colonies surrounded by a zone of hemolysis) and the SCV phenotype (colonies without pigmentation and hemolysis) should be isolated on solid media (**D1**,**D2**). Spontaneous reversion of the SCV phenotype into the NP should be noted (black arrow head, **D2**) throughout the complete diagnostic process. NP colonies (**D1**) should be used for usual diagnostic procedures (**F**), while SCV colonies (**D2**) should be identified by respective molecular approaches (**E**). Colonies of both types should be further processed for antimicrobial susceptibility testing (**H1**,**H2**), e.g., by disk diffusion test (DDT), which offers the advantage of revealing possible reversions, and for genotyping (**G**) to test the clonality of the different phenotypes. If possible, the SCV’s auxotrophism should also be determined on chemically defined media (CDM) agar plates (**I**) with disks supplemented with substances on which the growth of the SCV may specifically depend. Specific auxotrophism is visible as a growth zone around the respective disk (red arrowhead). All media for SCV cultivation should be incubated 48–72 h.

This review discusses the implications of the SCV phenotype status of staphylococci for cultivation, identification, verification, and the AST of SCVs and considers pre- and postanalytical factors (Figure 1).

## 2. Why Should We Specifically Look for Staphylococcal SCVs When Appropriate?

The first reason is due to the fact that SCVs are of specific clinical significance. They have been typically associated with chronic and relapsing infections [20]. Because they are often refractory to therapy, they often cause very long histories of suffering, often ranging from months to years [2].

Secondly, due to their intracellular persistence, they are protected from (i) immune defense mechanisms and (ii) antibiotics that cannot penetrate host cells. Moreover, the SCV phenotype itself may influence the susceptibility to antibiotic agents [21]. Thus, usual antibiotic treatment concepts may fail, and specific antibiotic strategies may be necessary, such as the inclusion of rifampicin as an intracellularly active agent and a longer treatment duration. To the contrary, some antibiotics or other agents may also induce or trigger the phenotypic switch into SCVs, as clinically reported, and experimentally used in the case of aminoglycosides and triclosan [22,23,24,25]. However, systematic studies for the optimal treatment of SCVs in the different types of infection are still lacking.

The third reason is that SCVs are hard to detect in routine microbiological diagnostics (as discussed below); thus, their real frequency and clinical significance may still be underreported.

## 3. When Should We Specifically Consider Staphylococcal SCVs in Diagnostics?

While the occurrence of SCVs during acute infections caused by *S. aureus* and other staphylococcal species has not been described, SCVs have typically been associated with patients with persistent and recurrent infections [20]. Frustrating disease courses that last from many months to years are typical [26,27]. Most studies and case reports on the recovery of SCVs from clinical specimens include foreign-body-associated infections, skin and soft-tissue infections, including diabetic foot ulcers, osteomyelitis and joint infections [2,4,28,29,30,31,32,33]. Thus, when chronic, relapsing and/or therapy-refractive courses are reported in such entities, a search for SCVs is specifically recommended. As mentioned above, extended exposure to aminoglycosides, e.g., gentamicin beads for the treatment of osteomyelitis, can also induce SCV formation in vivo and, thus should be included in the decision to initiate a special search for SCVs [25].

Persisting *S. aureus* SCVs are highly prevalent in patients with cystic fibrosis (CF), one of the most frequent hereditary diseases in the Caucasian population [34,35,36]. This autosomal recessive genetic disease is characterized by a highly viscous mucous layer, which impairs mucociliary clearance and leads to the development of an obstructive airway disease [37]. In the airways of CF patients, an age-dependent microbial infection pattern develops, with *S. aureus* being one of the first pathogens and successively replaced by *Pseudomonas aeruginosa* later in life [38,39]. The formation of the SCV phenotype in the CF lung environment is not restricted to one or a few distinct clones, but occurs in many different clonal lineages [40]. Of the CF lung-associated *S. aureus* SCVs, most are characterized as thymidine-auxotrophic [34,41]. To guide treatment and aid management, routine *S. aureus* SCV surveillance is recommended [2,42].

## 4. What Should Be Noted in the Preanalytics of SCV Detection?

As for many aspects of preanalytics, corresponding studies on SCVs are also lacking. Two issues might play a role here: (i) their adaptation to an intracellular lifestyle and (ii) their ability to spontaneously return to the normal phenotype. Naturally occurring SCVs from clinical specimens are particularly unstable and frequently revert to the normal phenotype during cultivation, sometimes immediately after striking out on solid media [43]. While natural revertants of clinical SCVs may also retain dominant protein features of the clinical SCV phenotype, they are visible to the naked eye on agar plates in the form of a classic colony morphotype [23].

Pathogens are unevenly distributed in the infected tissue. Thus, it is particularly important for SCVs that—if possible—multiple samples are obtained in the area of suspected infected tissue to minimize transport losses and increase sensitivity.

Based on the physiological characteristics of the SCV phenotype, the rapid transportation of specimens to the laboratory and beginning of cultivation is required. The usual specimen transport containers are sufficient.

## 5. What Should Be Considered during Cultivation of Staphylococcal SCVs?

The main diagnostic challenge is to recognize the tiny colonies and perceive them as possible representatives of species that normally grow in the form of larger colonies. This is, of course, particularly important in the case of *S. aureus*. It is not uncommon for *S. aureus* SCVs to be misinterpreted as commensal streptococci or corynebacteria and not processed further because the SCV colonies are nonhemolytic and nonpigmented (Figure 1). Further characteristics of staphylococcal SCVs are given in Table 1. Of note, the simultaneous presence of different, clonally identical SCV morphotypes, which differ in the graduation of their pigmentation and/or hemolysis and were recovered from the same or different specimens of a patient, has also been described [7,29].

**Figure 2:**
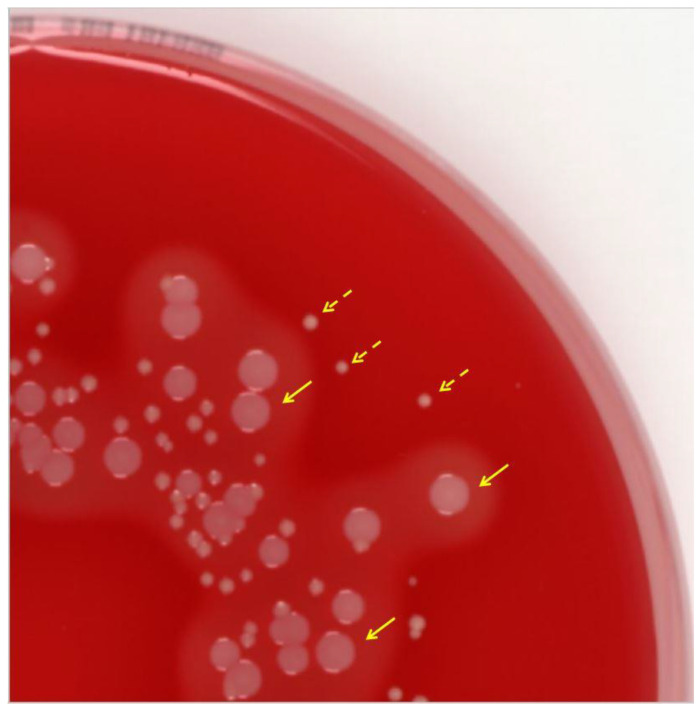
Columbia blood agar plate (with 5% sheep blood) showing *Staphylococcus aureus* after 24 h of incubation displaying the typical shapes of the normal *S. aureus* phenotype with grayish-pigmented colonies surrounded by a large hemolysis zone (solid arrows) and the *S. aureus* SCV phenotype with tiny, nonhemolytic, and nonpigmented colonies (dashed arrows). (Image has been processed to reduce irritating light reflections.)

SCVs have fastidious growth requirements [1]. While the normal staphylococcal phenotype is distinguished by medium-sized colonies that reach from 1 to 3 mm in diameter within 24 h, SCV colonies are maximally pinpoint in size and are approximately 1/10 the size of the parental strain (Figure 2). Often, they are not to be found at all after usual “overnight” incubation and need 2–3 days until the first colonies become visible. In the case of mixed flora, especially in enrichment cultures, this implies that they can also be quickly overgrown.

Another reason for the absence of SCVs in clinical samples is due to the instability of this phenotype. This means that, in the case of naturally occurring SCVs, subpopulations may spontaneously revert to the normal phenotype and may overgrow the SCVs. While this phenomenon is relatively easy to recognize with the naked eye on solid agar media, the phenotype switch is difficult to identify in broth media. The unstable SCV phenotype led to the in vitro generation of genetically defined knockout mutants displaying a stable SCV phenotype to study their metabolism, virulence, resistance development and other traits [23,45,46,47]. However, these mutants only partially reflect the features of clinical SCVs [23]. On the contrary, natural revertants of clinical SCVs have been shown to retain dominant protein features of the clinical SCV phenotype, which could potentially influence biochemical assays for diagnostic purposes [23].

Columbia blood agar plates are used in routine diagnostics for culturing staphylo-cocci, including SCVs. However, these and other routinely applied complex media contain substances on which they are specifically dependent (auxotrophic), such as menadione, hemin or thymidine, and even traces of these could induce a reversion to the normal phenotype [27]. Unfortunately, the required, specific, chemically defined media (CDM) are not commercially available and are themselves very laborious to produce [48].

Chromogenic agar media or other selective media (e.g., mannitol-salt-agar) may fail or not result, for example, in the expected phenotype of colored colonies [49]. In studies, the growth of SCVs was often not detected until after 72 h of incubation with these media [49].

To summarize the cultivation-related aspects, if SCVs are to be included in the diagnostic strategy, it is recommended that incubation must occur for at least 2–3 days. Inspection of the agar plates should be carried out at least daily and with the aid of a magnifying glass. These aspects represent a special challenge for the just-realized and future technical developments in microbiological diagnostics. In particular, automated laboratory systems must be programmed to incubate appropriate sets of agar plates for longer periods of time, and artificial intelligence-based systems must respond to divergent SCV morphologies.

## 6. How Can the Species of SCVs Be Determined?

The classical scheme of key biochemical/physiological characteristics for the identification of human staphylococcal species proposed by Kloos and Schleifer [50], as well as all subsequently developed semi-automated test systems based on biochemical features of the isolates, are to be regarded as unreliable for the identification of the species of clinical SCVs (Table 2). Due to the slow growth and changes in the metabolism of SCVs, usual biochemical reactions are strongly delayed or absent [51].

Systematic studies on the use of the matrix-assisted laser desorption/ionization time-of-flight (MALDI-TOF) mass spectrometry (MS) for the identification of staphylococcal SCVs are not available. However, if enough SCV biomass is available, this approach is feasible for routine purposes (author’s own experience). This can be explained, since the ribosomal proteins necessary for spectra generation in diagnostically used MS devices are also proportionally dominant in SCVs. A paper written in Japanese showed, for thymidine-dependent SCVs, that both the direct transfer-formic acid MS sample preparation method, as well as the ethanol-formic acid method, yielded equivalent results [52]. Some case reports about non-staphylococcal SCVs also indicate the suitability of the MALDI-TOF MS approach [53,54].

Since fluorescence in situ hybridization (FISH) method has left the experimental stage [55], this approach might be a promising tool for the future detection of SCV-infected host tissues. The general applicability of this method has already been shown by the use of a 16S rRNA-directed in situ hybridization technique with fluorescence-labelled oligonucleotide probes specific to *S. aureus* for tissue samples from a patient with a brain abscess caused by *S. aureus* SCVs [56].

Depending on the bacterial load, all nucleic-acid-based methods directed to the respective target genes are, in principle, capable of directly detecting infections by staphylococcal SCVs in tissues or identifying SCV colonies. However, a distinction between both phenotypes is not possible with standard tests (see below for the identification of SCV-formation-inducing mutations). Phenotype-detecting molecular approaches are not described at present, but conceivable, e.g., on the basis of a reverse transcription polymerase chain reaction (RT-PCR). However, the diversity of the SCV phenotype and its underlying mechanisms make it difficult to find a universal approach or cover all variants.

Nucleic acid-based molecular approaches, in particular sequencing approaches, are the ultimate tools to verify the species of SCV isolates. In a 16S rRNA partial sequencing study including staphylococcal SCVs, the sequencing approach was unambiguously able to identify them correctly at the species level while two phenotypic methods (API ID 32 Staph and VITEK 2) misidentified the SCVs [51]. Other universal or specific target genes that have been shown to be sufficiently discriminatory for staphylococcal identification [51,57,58,59,60] may also be used for the species identification of SCVs.

## 7. How Can the SCV Phenotype Be Objectified and Characterized?

**Figure 3:**
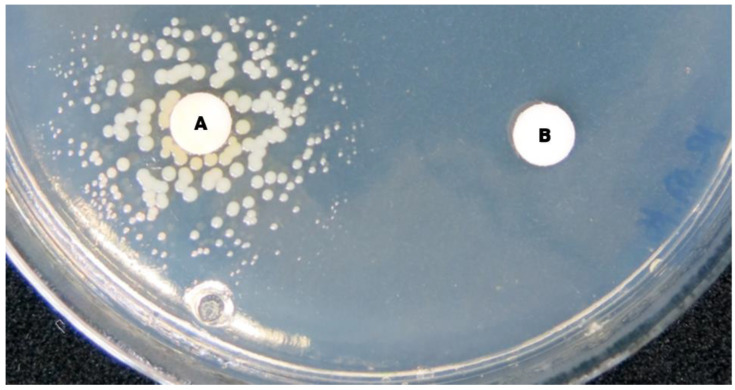
Agar plate for auxotrophy testing of an *S. aureus* SCV after 48 h of incubation with disks impregnated with substances (A, B) on which the growth of an SCV may specifically depend. An SCV suspension was streaked on the entire agar surface. While substance A resulted in the growth of the SCV in the diffusion zone around the left disk with partial reconstitution of the normal phenotype (note size and pigmentation), no growth can be observed around the right disk impregnated with substance B.

The typical inability of bacterial cells exhibiting the SCV phenotype to synthesize organic compounds required for their growth, i.e., their auxotrophy, can best be tested by the use of surface-dried (condensation- and water-free) CDM agar and blank antimicrobial susceptibility disks supplemented with an array of those substances known to most frequently be necessary for staphylococcal auxotrophs, such as hemin, menadione and thymidine (Figure 3) [61,62]. If the test result is negative, other substances could be considered as well, e.g., oil derivates and fatty acids (oleic acid, tween 80, polyethylene glycol and others) [17]. It is worth testing different concentrations of the respective substances. To test for CO_2_-dependent SCVs, the plates could be incubated aerobically with and without 5% CO_2_ supplement [63]. However, it will not be possible to determine the underlying auxotrophy in all cases.

To characterize a microbial phenotype in a comprehensive and objectifiable manner, a phenotypic method based on molecular structures would be ideal. The Fourier-transform infrared spectroscopy (FT-IR), a non-destructive technique, meets these requirements and was shown to allow for the rapid characterization of structural characteristics of biological molecules, as well as more complex materials such as host cells and microbes [64,65]. For staphylococcal SCVs, FT-IR proved to be a real-time readout tool that could be used to discriminate different staphylococcal phenotypes under liquid culture conditions [43]. Remarkably, the FT-IR spectra of SCVs with different genetic backgrounds were much more similar to one another than the spectra from any of their corresponding parental strains, further highlighting the significant differences between the two phenotypes revealed by proteomic and metabolomic studies [23,46,66]. As FT-IR is increasingly used for strain-typing for hospital hygiene and epidemiologic purposes, it should be noted in this context that phenotypic variants might be misclassified despite sharing a genetic background with corresponding wildtype isolates.

In search of a genetic basis for the different auxotrophic, naturally occurring types of SCVs and those resulting from experiments to construct stable genetically defined mutants displaying the SCV phenotype, several gene loci with mutations within the menaquinone, heme and thymidine pathways have been identified, including *menB*, menD, *hemA*, *hemB*, *hemG*, *hemH*, *cta* and *thyA* [16,45,67,68,69,70,71,72]. Moreover, several other auxotrophies and dependencies (e.g., fatty-acid and CO_2_) and mechanisms (e.g., non-protein-encoding RNAs, oxidative and stringent stress response) inducing the SCV formation have been described [17,30,73,74,75,76,77]. Whereas in the past it would have taken an enormous amount of time and effort to search for and identify these potential underlying alterations, the recent next-generation tools for whole-genome sequencing (WGS) may significantly speed up this process and make its use for diagnostic purposes more realistic in the future in the case of mutation-based SCV formations.

When different phenotypes (normal and one or more SCV phenotypes) have been isolated, genotyping the different morphotypes is advisable to assess their clonality. The use of WGS, e.g., as whole-genome multilocus sequencing typing (wgMLST), would have the additional advantage of allowing for the determination and comparison of the genotype with parental isolates, if available. However, the classical sequencing-based genotyping techniques, such as multilocus sequence typing (MLST) using housekeeping genes and *spa* typing (only for *S. aureus* SCVs), are also applicable [78,79,80,81]. They have replaced the more laborious and difficult-to-analyze techniques, such as the banding pattern-based pulsed-field gel electrophoresis (PFGE), which were used in earlier decades of SCV research [34,82].

## 8. Is It Necessary to Test SCVs Separately for Resistance Determination and How Can This Be Performed?

First of all, it is necessary to emphasize a special feature of the SCVs: their intracellular location protects them from the action of all antibiotics that cannot penetrate the membrane of the host cells and the therapeutic implications that result from this phenomenon; for further details, refer to the literature [2,21]. Although this aspect is not part of routine AST, it should be part of the laboratory findings (please, see below) to alert the treating physician that agents that have tested susceptible in vitro may not be effective in the case of SCVs due to this type of functional resistance.

While SCVs and their corresponding normal phenotypes show similar categorizations in AST for most antibiotics, the altered metabolism subsequent to the phenotypic switch may lead to regular changes in the susceptibility to distinct antibiotics, including antifolate agents and aminoglycosides [32,83,84]. The activity of cell-wall-active antibiotics may be reduced in SCVs, possibly due to their low growth rate and reduced cell division [85,86]. Apart from the known metabolic changes and their effects, SCV isolates may have reduced resistance compared to their parent strains [21,87]. However, intracellular SCVs were shown to be hypersusceptible to β-lactams in a cell culture model based on the interaction of acidic pH and oxidant species in vacuoles [88]. Therefore, altered antibiotic susceptibilities and resistances, respectively, should be considered, which necessitates the separate determination of the susceptibilities of SCV isolates recovered from clinical specimens.

Unfortunately, the usual time- and density-influenced, culture-based AST approaches are influenced by the different physiology of the SCV phenotype, especially by the delayed growth rate. At present, no standardized protocols for manual assays or (semi) automated testing procedures exist. Nucleic acid amplification tests, e.g., for molecular methicillin resistance detection, are relatively easy to perform. PCR or isothermal amplification methods additionally require a drastically increased number of colonies (or measurement of DNA concentration for exact test adjustments) [89]. The same applies for the use of an anti-PBP2a slide latex agglutination assays in terms of the required colony number [89]. Routine culture-based AST methods were developed for fast-growing bacteria and are, therefore, difficult or impossible to perform and interpret for SCVs. SCVs have been tested by agar-based techniques such as the agar diffusion method or gradient tests (Etests). Deviating from the usual procedure, the SCVs should be cultivated for several days on the agar plates until they are clearly visible. Incubation with CO_2_ may be beneficial. Nevertheless, the readout of the agar plates is often challenging, and the results are often ambiguous. Broth-based methods may pose the risk of spontaneous phenotype reversion to the normal phenotype being overlooked.

In future, intracellular AST of SCVs in host cells (e.g., monocytes and nonprofessional phagocytes) might be an alternative approach that better reflects in vivo conditions, and promising cell culture models have already been developed [90,91].

## 9. How Should an SCV Finding Be Reported, Commented on and Managed?

Apart from respiratory specimens of CF patients, SCVs are rather rarely reported in microbiological routine diagnostics and, thus, are often little known or unknown to clinicians. Thus, the identification of SCVs and respective AST findings should not be reported without further commentary. An additional phone call to the attending physician would be preferable to avoid overlooking or misinterpretating this special finding, which should be avoided as the detection of SCVs may result in specific therapeutic consequences due to their specific features and intracellular location, as described above.

Consequently, comments for the clinician in the case of SCV findings should include statements about (i) their nature as a phenotype that renders staphylococci an intracellular pathogen, (ii) their typical association with chronic and relapsing infections, (iii) the non-standardized antibiotic testing performed and the influence of the functional resistance of SCVs, and (iv) possibly deviating infection management.

Finally, the calamitous combination of chronic and relapsing course of infection, difficult detection and limitations in treatment necessitates a coordinated approach to clinicians, microbiologists and other disciplines, e.g., pharmacists and pathologists, to successfully manage SCV-related infections. These requirements could be met by an integrated stewardship model comprising antimicrobial, infection prevention and diagnostic stewardship, as proposed elsewhere [92].

## Figures and Tables

**Table 1:** Key colony morphological characteristics of staphylococcal SCVs compared to the normal phenotype.

Colony Morphological Characteristics *	SCV Phenotype **	Normal Phenotype ***
Colony appearance	Pinpoint or “fried-egg”-like	Smooth, raised and glistening colonies with entire margins
Size after 24 h of incubation	Invisible or tiny (ca. 1/10 of the normal phenotype)	1–3 mm or larger
Pigmentation	Not present or reduced	Unpigmented or coloured, varying from grey to yellow–orange
Hemolysis	Not present or reduced	Weak to strong zone of β-hemolysis (in particular, *S. aureus* and *S. haemolyticus*) or without hemolysis (most of the CoNS species)

* Note that species- and strain-dependent characteristics may occur (modified acc. [2]); ** Indicated characteristics compared to isolates of the same staphylococcal species exhibiting the normal phenotype; *** For the normal phenotype, those of the most prevalent staphylococcal species encountered in human specimens are preferentially summarized, as described in Bergey’s Manual of Systematic Bacteriology [44].

**Table 2:** Key features for the identification and characterization of staphylococcal SCVs compared to the normal phenotype.

Feature *	SCV Phenotype	Normal Phenotype (NP)
Growth rate	Reduced ** (approx. 48 to >72 h)	Normal (6 to 24 h)
Auxotrophism	Present (different types; most frequent hemin-, menadione-, thymidine-dependent SCVs, less often dependency on fatty acids, CO_2_ and other factors)	Absent
Fermentation and other biochemical reactions	(Strongly) delayed to absent	Normal (“overnight”)
Coagulase activity ***	(Strongly) delayed	(2) 4 to 24 h
Catalase activity	(Strongly) delayed	Rapid reaction

* Note that species- and strain-dependent characteristics may occur; ** approximately determinable by observation of colony growth, objectifiable by growth curve analysis; *** in the case of coagulase-positive/variable staphylococcal species.

## Data Availability

Not applicable.
